# Sustainable Urbanization Performance Evaluation Based on “Origin” and “Modernization” Perspectives: A Case Study of Chongqing, China

**DOI:** 10.3390/ijerph15081714

**Published:** 2018-08-10

**Authors:** Jing Bian, Hong Ren, Ping Liu, Yu Zhang

**Affiliations:** 1 School of Construction Management and Real Estate, Chongqing University, Chongqing 400045, China; bianjing125@126.com (J.B.); zhangyu_0112@foxmail.com (Y.Z.); 2 School of Civil Engineering, Lanzhou University of Technology, Lanzhou 730050, China; liupvip@foxmail.com

**Keywords:** sustainable, urbanization performance evaluation, index system, multilevel extension method, origin, modernization

## Abstract

Although the acceleration of urbanization brings social and economic development, it also produces various problems. Accurate evaluation of sustainable urbanization performance can help local governments summarize experiences and solve problems. Sustainable urban development should focus not only on modern construction, but also on original natural ecosystems and traditional cultural protection. This paper develops a holistic framework based on an “origin” and “modernization” perspectives and uses the multilevel extension method and the Analytic Hierarchy Process (AHP) method for accurately evaluating sustainable urbanization performance. A case study of Chongqing City in China demonstrates the process of using the holistic framework and evaluation method. The empirical results from this study indicate that Chongqing has a medium level of sustainable urbanization. The city is considered as a medium level in “origin” performance and the “modernization” performance is good, while uncoordinated. The case study reveals that the proposed framework and the method are effective theoretical bases for policy-makers and practitioners to understand the performance of urban sustainability and for promoting urbanization toward better sustainability. Beyond the application case, the holistic framework and method can be applied to other cities.

## 1. Introduction

The rate of urbanization in the world has increased by 21% and, during the past 60 years, more than 50% of the world’s population has concentrated in urban areas [1]. Since 1978, when China began the reform and opening-up process, urbanization progressed rapidly [2,3,4,5,6]. By the end of 2017, China’s urbanization rate had reached 58.52% [7] and is projected to climb to 75% by 2050 [8]. Urbanization brings many benefits, such as accelerated economic development, and improved quality of living standards [9,10,11,12,13,14]. However, a series of issues have arisen, such as environmental pollution, cultural issues, urban housing shortages, and traffic congestion, all of which lead to social problems, unbalanced regional economic development and more [15,16,17,18].

Many governments and institutions around the world have tried various strategies and policies to promote sustainable urban development. UN-Habitat implemented the Urban Management Program to enhance the urban management capacity to reduce urban population poverty and the impact of natural disasters [19]. The Mexican government produced the Mexico City Green Plan in 2007 [20]. The Melbourne government launched Melbourne’s City Plan 2010 [21]. Urbanization contents in China’s sustainable development have been enriched too. The Chinese government issued Ten Strategic Policies for Environment and Development in 1993 [22]. China also promulgated China’s Agenda 21 in 1994, which was a blueprint for China’s implementation of the sustainable development strategy. China’s National Development and Reform Commission issued, in March 2014, the “National New-Type Urbanization Plan (2014–2020)” [23], which paid more attention to the quality of urbanization [24,25]. Under such challenges, the top priority is to develop an effective index system and a scientific method to evaluate the sustainable urbanization performance. The purposes of the sustainable urbanization performance evaluation include promotion impact and comparative effect, which provide policy makers with suggestions [26]. Therefore, evaluating the sustainable urbanization performance is a matter of significance.

Aligned with this need, researchers have been concerned with sustainable urbanization performance evaluation [4,6,9]. Shen et al. [27] introduced an elasticity coefficient model to evaluate sustainable urbanization by measuring the relationship between urbanization and urban sustainability. Zhou et al. [6] assessed the urbanization performance from a perspective of structure and function analyses by using the entropy weight method. Shen et al. [9] evaluated sustainable performance from a global perspective by employing environment, economic and social dimensions. Li et al. [28] developed a full permutation polygon synthetic indicator method to analyze the performance of economic growth and efficiency, ecological and infrastructural construction, environmental protection, and social and welfare progress dimensions. Zhao et al. [26] established an information entropy model by using five systems, namely urban construction, economic development, social development, ecological environment and urban rural development dimensions. Gao et al. [3] analyzed the urbanization levels based on the defense meteorological satellite program (DMSP) nighttime light data in China from 1992 to 2012. Jiao et al. [29] used a structural equation model to assess China’s urbanization performance by employing four aspects, namely economic, social, environmental and resource. Mori and Yamashita [30] provided a City Sustainability Index (CSI) framework to assess the urban sustainability, which included environmental, economic and social dimensions. Xu and Coors [31] used system dynamics model, GIS and 3D visualization techniques for evaluating the urban sustainability. Shen et al. [32] identified an International Urban Sustainability Indicators List based on the examination of nine different practices, where sustainability indicators were divided into environmental, economic, social and governance dimensions.

However, as mentioned above, there are two limitations in evaluating both the sustainable urbanization performance index and methods: (1) Many previous studies focused on the modernization of the urban construction index system, but few frameworks focused on original natural ecosystems and traditional cultural protection for the sustainable urbanization performance index. However, sustainable urban development should focus not only on modern construction, but also on the original elements of the city. The study by Raymond et al. proposed that natural ecosystems provide feasible solutions to solve various challenges, for instance, climate change, disaster prevention, water resource protection and sustainable urbanization [33]. Concurring with this, Keesstra et al. [34] showed that nature based solutions have superiority in improving the sustainability of river basin systems by promoting soil and landscape functions. Friedmann [35] proposed that urbanization not only concentrated on population, non-agricultural activities and the change of regional landscape, but also emphasized the diffusion of urban culture, urban lifestyle and values. Therefore, it is important to accurately evaluate the sustainable urbanization performance from “origin” and “modernization” perspectives. (2) The existing evaluation methods have some applicable limitations. The determination of weights in the entropy method depends on the samples, which is restricted in application, for example. The determination of the index vector in the fuzzy comprehensive evaluation method is subjective. Data envelopment analysis is sensitive to outliers. DMSP data lacks information beyond the light data, which is limited to the detection of human activities in unlighted areas. Recently, the developed multilevel extension evaluation method can overcome the shortcomings of the above methods. It has been successfully applied in many fields [36], but rarely in the sustainable urbanization performance evaluation. The multilevel extension method is therefore applied in this paper. 

Considering the limitations of the existing index and methods, the objectives in this study are therefore to: (1) construct a comprehensive urbanization index system based on “origin” and “modernization” perspectives, and (2) to propose the multilevel extension model to evaluate the sustainable urbanization performance. The remainder of this paper is organized as follows: Section 2 elaborates on the screening of indicators based on “origin” and “modernization” perspectives for sustainable urbanization performance evaluation. The Analytic Hierarchy Process (AHP) method and the multilevel extension method are introduced in Section 3. Section 4 applies these methods to identify the sustainable urbanization performance of a case study of Chongqing in China. Based on the results from Section 4, the outcomes are further discussed in Section 5. Finally, conclusions are presented in Section 6.

## 2. Index System of Sustainable Urbanization Performance Evaluation 

**Figure 1:**
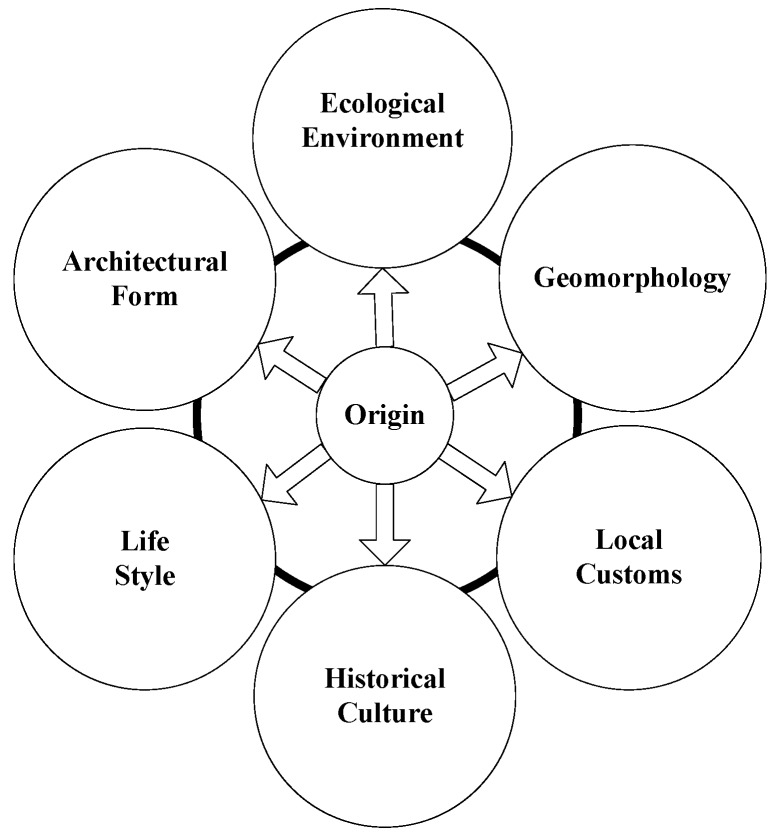
The connotation of “origin”.

The framework of sustainable urbanization based on “origin” and “modernization” perspectives is proposed [37] in this paper. The concept of “origin” is a people-oriented development basis, which is not corrupt or poor quality. It includes not only a variety of beautiful and natural environments, but also a variety of harmonious human environment. It can be considered that “origin” is the fusion of nature and traditional culture, meaning that humans are in harmony with nature and culture. Figure 1 illustrates the connotation of “origin”.

**Figure 2:**
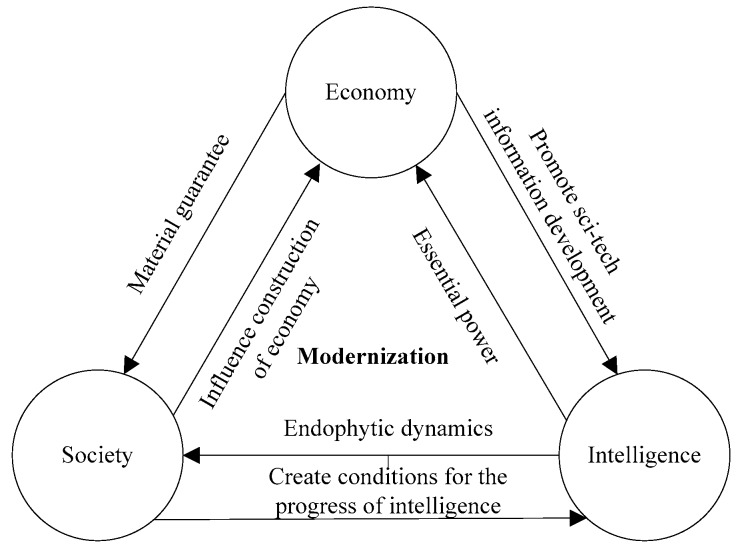
The connotation of “Modernization”.

Modern construction technology, materials, advanced equipment and facilities are the bases for improving the safety, durability, and economy of urban areas. The concept of “modernization” is an organic unity of the “economy-society-intelligence (science and technology information)” mindset, as shown in Figure 2. The concept of “origin” and “modernization” means that humans not only enjoy a developed and conveniently modernized society, but also enjoy a comfortable and cozy life given by the natural environment and rich culture.

A complete index system is a crucial task to evaluate the sustainable urbanization performance quantitatively [38]. According to relevant research [9], the determination of the index system is established on the principles of maturity, measurability, independence and operability. The framework was developed based on “origin” and “modernization” perspectives. First, a comprehensive literature review is conducted and a preliminary list of urbanization indicators is formed. Second, preliminary indicators are selected based on the expert interview method. Five relevant scholars and nine government regulators are invited to be interviewed. Following repeated discussions, the experts reached agreement that all urbanization indicators were reasonable and meaningful. Finally, a four-level index system with 23 indicators is constructed, as shown in Table 1.

## 3. Materials and Methods

### 3.1. Weight Determination by Analytic Hierarchy Process (AHP) Method

The analytic hierarchy process (AHP) method, developed by Saaty in the 1970s, is an effective decision-making method with multiple criteria [39]. AHP can solve multi-criteria decision issues and avoid inconsistencies decision-making, which is a system synthesis method. Because the evaluation index system has many indicators with each level, AHP is used to determine the weights of indicators in the urbanization performance evaluation. The calculation procedures are as follows:

(1) Clarify the problem and build a hierarchical structure, as shown in Equation (1):
  (1) $$C=\left[\begin{array}{cc} \begin{array}{cc} c_{11} & c_{12}\\ c_{21} & c_{22} \end{array} & \begin{array}{cc} \cdots & c_{1n}\\ \cdots & c_{2n} \end{array}\\ \begin{array}{cc} \vdots & \vdots \\ c_{n1} & c_{n2} \end{array} & \begin{array}{cc} \ddots & \vdots \\ \cdots & c_{nn} \end{array} \end{array}\right]$$
(2) Construct pairwise comparison matrices: AHP makes pairwise comparison on the importance of the target. The importance scale suggested by Saaty [39] is used to indicate the relative importance of the indicators, as shown in Table 2.
(3) Calculate the weights: the mathematical process commences to normalize and calculate the relative weights for each matrix. The relative weights are given by the right eigenvector (ω) corresponding to the largest eigenvalue:
  (2) $$C\omega =\lambda_{max}\omega ,\;\overset{n}{\underset{i=1}{\sum }}\omega_{i}=1$$
  where ω is the eigenvector, $\lambda_{max}$ is the largest eigenvalue of C, $\omega_{i}$ is the eigenvalue of the given matrix.
(4) Check the consistency of the judgment matrix: the consistency index (CI) for matrix size is calculated from the correlation values [39]:
  (3) $$\mathrm{Consistency}\;\mathrm{index}\;\left(\mathrm{CI}\right)=\frac{\lambda_{max}-n}{n-1}$$
  (4) $$\mathrm{Consistency}\;\mathrm{ratio}\;\left(\mathrm{CR}\right)=\frac{\mathrm{CI}}{\mathrm{Random}\;\mathrm{index}\;\left(\mathrm{RI}\right)}$$
  where RI is the random consistency index related to the dimension of matrix, as shown in Table 3.

If CR ≤ 0.10, the matrix C satisfies the consistency constraint. Otherwise, the judgment matrix needs to be adjusted.

### 3.2. Multilevel Extension Method

The multilevel extension method was employed for evaluating the sustainable urbanization performance in this study. The extension theory, originated by Cai [40], can solve contradictions and incompatibility problems. Compared with the existing urbanization performance evaluation method, this method has the following advantages: (1) it can overcome the inherent disadvantages of ambiguity and uncertainty in traditional evaluation methods and make up the restrictions of other methods in missing information [41], (2) it can quantify the qualitative indicators and also can be applied to assess the fuzzy models [36], (3) it can determine which comprehensive level of sustainable urbanization is closer to which grade by eigenvalue of grade variable [42], (4) it is suitable to solve multiple indicator evaluation problems [43], which is easy to calculate and can also be implemented on the computer [44]. It is an objective and effective method for evaluating the sustainable urbanization performance. The basic steps of the multilevel extension method are as follows [36,45].

#### 3.2.1. Determination of Classical Domain, Joint Domain

Matter element is the basic logic cell in the extension method. Name a matter N, and its value V about a characteristic C. The group R = (matter, characteristic, value) = (N,C, *V*) are the three key elements to describe a matter: R is called a matter-element; N means the standard grades of the sustainable urbanization performance evaluation; C means the sustainable urbanization indicators. The sustainable urbanization indicators are divided into n types that is $C=\left\{C_{1},\;C_{2},\cdots ,C_{n}\right\}$, supposing that each indicator $C_{i}\;\left(i=1,2,\cdots n\right)$ below with an $n_{i}$ sub-indicator that is $C_{i}=\left\{C_{i1},\;C_{i2},\cdots ,C_{in}\right\}$, where $C_{ik\;}\left(k=1,2,\cdots ,n_{i}\right)$ is the *k*th sub-indicator of the *i*th subset.

(1) The classical field:
  (5) $$R_{j}=\left(N_{j},\;C_{ik},\;V_{kj}^{\;}\right)=\left[\begin{array}{ccc} N_{j} & C_{11} & \langle a_{1j,}^{1}b_{1j}^{1}\rangle \\ & \vdots & \vdots \\ & C_{ik} & \langle a_{kj,}^{i}b_{kj}^{i}\rangle \\ & \vdots & \vdots \\ & C_{nn_{i}} & \langle a_{n_{i}j,}^{n}b_{n_{i}j}^{n}\rangle \end{array}\right]$$
  where $N_{j}=\left(j=1,2\cdots m\right)$ is the divided grade j, $C_{ik}$ is the j-th characteristic of matter-element, $V_{kj}^{i}=\langle a_{kj}^{i},b_{kj}^{i}\rangle$ is the classical field, which is the stipulated value range of $N_{j}$ about $C_{ik}$.
(2) The segment field:
  (6) $$R_{p}=\left(N_{p},\;C_{ik},\;V_{ip}\right)=\left[\begin{array}{ccc} N_{p} & C_{11} & \langle a_{1p,}^{\;}b_{1p}^{\;}\rangle \\ & \vdots & \vdots \\ & C_{ik} & \langle a_{ip,}^{\;}b_{ip}^{\;}\rangle \\ & \vdots & \vdots \\ & C_{nn_{i}} & \langle a_{np,}^{\;}b_{np}^{\;}\rangle \end{array}\right]$$
  where $N_{p}$ is all evaluation grades for the sustainable urbanization performance, $V_{ip}=\langle a_{1p,\;}b_{1p}\rangle$ is the segment field, which is the stipulated value range of $N_{P}$ about $C_{ik}$.
(3) Determine the matter-elements:
  (7) $$R=\left[\begin{array}{ccc} P & C_{11} & v_{11}\\ & \vdots & \vdots \\ & C_{ik} & v_{ik}\\ & \vdots & \vdots \\ & C_{nn_{i}} & v_{nn_{i}} \end{array}\right]$$
  where *P* is the matter element to be evaluated, $C_{ik}$ is the characteristic of *P*, $v_{ik}$ is the value of *P* about $C_{ik}$, namely the actual value of the evaluated index.

#### 3.2.2. Calculation of the Correlation Degree

First, calculate the dependent degree that $C_{ik}$ about each grade j for the sustainable urbanization performance evaluation:

(8) $$K_{kj}^{i}\left(v_{ik}\right)=\{\begin{array}{cc} \frac{\rho \left(v_{ik},V_{kj}^{i}\right)}{\rho \left(v_{ik},V_{ip}\right)-\rho \left(v_{ik},V_{kj}^{i}\right)} & x\notin \left(a_{kj}^{i},b_{kj}^{i}\right)\\ 0.5 & v_{ik}=a_{kj}^{i}\;\mathrm{or}\;v_{ik}=b_{kj}^{i}\\ -\frac{\rho \left(v_{ik},V_{kj}^{i}\right)}{b_{kj}^{i}-a_{kj}^{i}} & x\in \left(a_{kj}^{i}.b_{kj}^{i}\right) \end{array}$$

where $\rho \left(v_{ik,\;}V_{kj}^{i}\right)$ is the distance between the point $v_{ik}$ and the interval $V_{kj}^{i}$; where the formula of distance between the point and the interval 〈a,b〉 is:

(9) $$\rho \left(x,\langle a,b\rangle \right)=\left|x-\frac{\left(a+b\right)}{2}\right|-\frac{b-a}{2}\;$$

Then, we calculate the correlation degree $K_{ij}\left(v_{i}\right)$ and $K_{j}\left(P\right)$:

(10) $$K_{ij}\left(v_{i}\right)=\overset{n_{i}}{\underset{k=1}{\sum }}\omega_{ik}K_{kj}^{i}\left(v_{ik}\right)$$

(11) $$K_{j}\left(P\right)=\overset{n}{\underset{i=1}{\sum }}\omega_{i}K_{ij}\left(v_{i}\right)$$

#### 3.2.3. Grade Judgment

If:

(12) $$K_{j_{o}}(P)=\underset{j\in \{1,2,\cdots ,m\}}{\max}K_{j}(P)$$

then the object of P belongs to grade $j_{0}$ and:

(13) $$\bar{K_{j}(P)}=\frac{K_{j}(P)-\underset{j\in \{1,2,\cdots ,m\}}{\min}K_{j}(P)}{\underset{j\in \{1,2,\cdots ,m\}}{\max}K_{j}(P)-\underset{j\in \{1,2,\cdots ,m\}}{\min}K_{j}(P)}$$

then the eigenvalue of grade variable $j^{*}$ of the evaluated object P is:

(14) $$j^{*}=\overset{m}{\underset{j=1}{\sum }}j\bar{K_{j}\left(P\right)}/\overset{m}{\underset{j=1}{\sum }}\bar{K_{j}\left(P\right)}$$

$j^{*}$ can indicate the degree to which the evaluation result tends to be biased.

### 3.3. Overall Research Method

**Figure 3:**
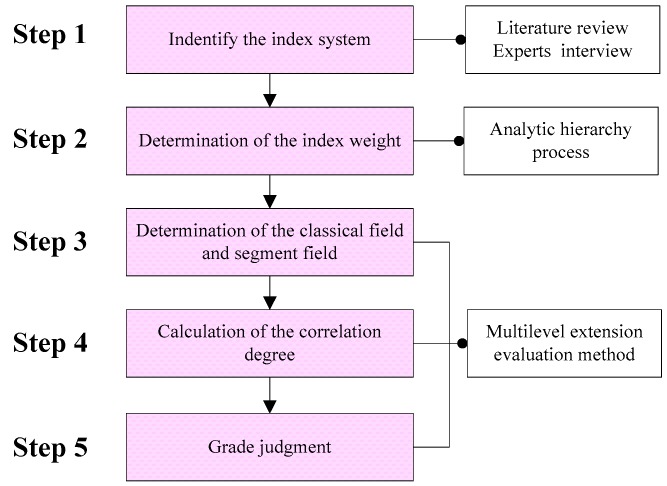
Flowchart of the method in this study.

A flowchart of the overall methodology combining the AHP method and the multilevel extension method adopted here is presented in Figure 3. First, literature review and experts interview methods were used to construct the index system. Second, the AHP method was used to determine each index weight. Then, the multilevel extension evaluation method was employed to evaluate the sustainable urbanization performance.

### 3.4. Study Area and Data Collection

#### 3.4.1. Study Area

**Figure 4:**
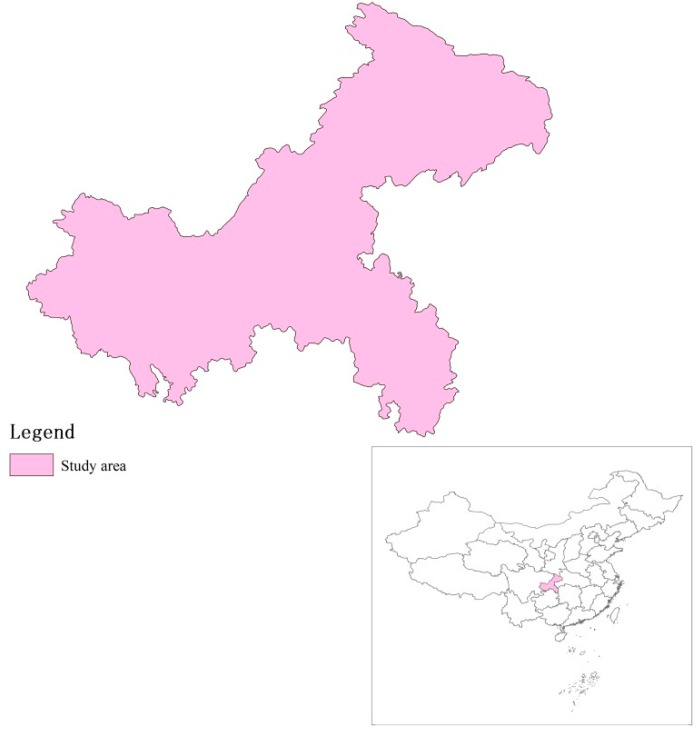
Location of the study area.

Chongqing is the only city under the central government in the west of China with a total area of 82,402 km^2^, as shown in Figure 4. Chongqing is surrounded by rolling green mountains, and is located on the Yangtze and Jialing Rivers. The population in Chongqing in 2016 reached 33.92 million [46]. The gross domestic production (GDP) of Chongqing was about 1.76 trillion Yuan in 2016 [46]. Chongqing’s economy and society have undergone a great change since it became the municipality city in 1997. Chongqing is also a city with a favorable natural environment and local folk culture. Therefore, this paper selects Chongqing as a case study for evaluating the sustainable urbanization performance. 

#### 3.4.2. Data Collection

The required data of Chongqing in 2015 were gathered for this study. The actual values were from statistical data, evaluation reports and documents issued by the state. The statistical data were derived from “China Statistical Yearbook, 2016” [47] and “China Statistical Yearbook on Science and Technology, 2016” [48]. Evaluation reports were collected from “Study on the Evaluation of Ecological Environment Quality in Chongqing” [49], “Evaluation of Chinese Cities Basic Public Service Capability” [50]; “Evaluation Report of the Level of Information Development in China” [51]. Documents issued by the state were obtained from the websites of “National data of China” [52], “The First to Seventh Batches of Key National Heritage Conservation Units” [53] and “The First to Fourth Batches of National Intangible Cultural Heritage Lists” [54].

## 4. Results 

### 4.1. Weights of Indicators 

The AHP method was used to calculate the weights of indicators. Nine experts were invited to make decisions, including five university professors, and four from a government administrative department. The weights of indicators were determined as follows by using Equations (1)–(4). The results are shown in Table 4, Table 5, Table 6, Table 7, Table 8, Table 9, Table 10, Table 11, Table 12 and Table 13.

### 4.2. Determination of the Classical Field and Segment Field 

The classical field, segment field, actual value and data source are shown in Table 12. The ranges of classical fields and segment fields were considered using the relevant literature [50,51,55] and experts discussion. The sustainable urbanization performance was divided into five grades, where the five rankings of “excellent”, “good”, “medium”, “fair”, “poor” were assigned scores of 5, 4, 3, 2, and 1.

### 4.3. Calculation of the Correlation Degree and Grade Judgment 

The AHP was used to calculate each indicator weight. The correlation values were calculated by Equations (8)–(14). Thus, the results of the comprehensive sustainable urbanization performance and the sustainable urbanization performance of sub-index for Chongqing were obtained (see Table 13). According to Table 13, the comprehensive urbanization performance of Chongqing was at medium level ($j_{0}=3$), and $j^{*}=3.411$ indicates the comprehensive urbanization performance was between a good and medium level, but closer to medium level.

### 4.4. Sensitivity Analysis

**Figure 5:**
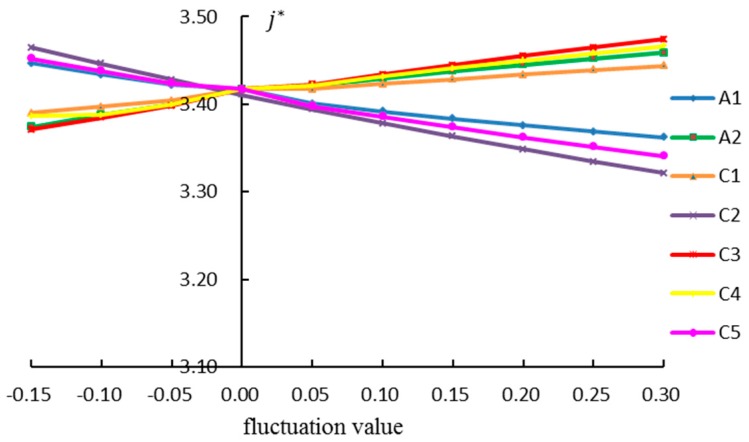
Sensitivity analysis of the weights of sustainable urbanization index (A1 origin; A2 modernization; C1 nature; C2 traditional culture; C3 economy; C4 society; C5 intelligence).

Sensitivity analysis was performed based on the sustainable urbanization index system. Figure 5 shows the results when the weights of the sustainable urbanization index were changed by ±10% and ±15%.

According to Figure 5, the comprehensive sustainable urbanization performance was correlated positively to the weights of A2, C1, C3 and C4. The weight of C3 is the most sensitive. The comprehensive sustainable urbanization performance was correlated negatively to the weights of A1, C2 and C5, with the weight of C2 the most sensitive. The sensitivities of the weights of sustainable urbanization index for A1 and C1 were weak.

Considering the above sensitivity analysis, the authors concluded that the weights of C2 and C3 were sensitive in the sustainable urbanization indices. Regarding the sustainable urbanization management process, these indices should be analyzed mainly to improve the sustainable urbanization performance of Chongqing. Allowing for weight sensitivity analysis, it was seen that the sensitivity of the index weights were relatively small, which shows the solution is implementable and robust. 

## 5. Discussion 

### 5.1. Sustainable Urbanization Performance Evaluation Analysis

**Figure 6:**
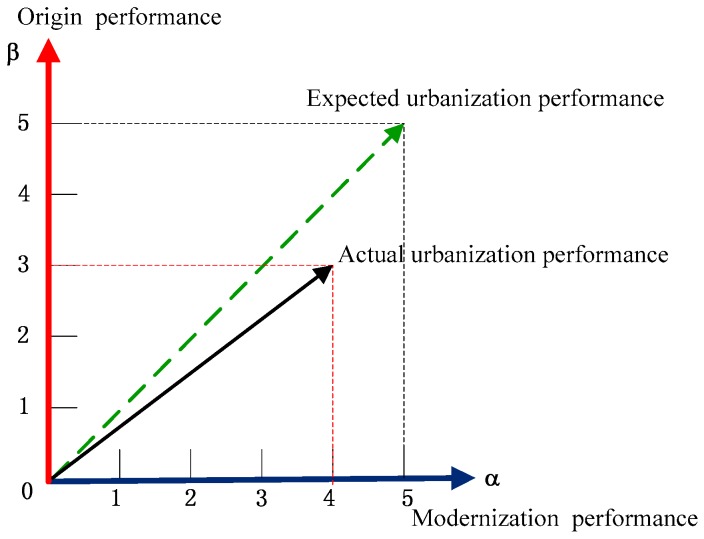
The sustainable urbanization performance based on “origin” and “modernization” perspectives of Chongqing.

According to Figure 6, the actual urbanization performance deviated from the expected urbanization performance, and the origin performance was medium ($j_{0}=3$) and the while modernization performance was good ($j_{0}=4$), both were uncoordinated. The past decade witnessed Chongqing’s rapid growth, with the accompanying pollution and congestion in Chongqing particularly serious [56]. The study by Zhang [57] explained that rapid urbanization led to increased ecological pressure within the Chongqing metropolitan area due to a concentration of the population within the central city. Chongqing’s unique geographic features and natural environment advantages are doomed to its own distinct development path. Consequently, urban managers should strike a right balance between “origin” and “modernization” and lead Chongqing to create a livable city.

**Figure 7:**
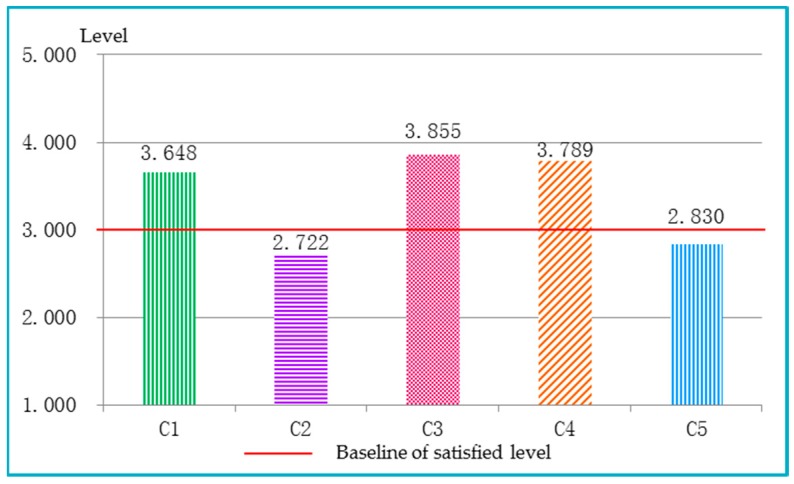
The sustainable performance of five subsystems in Chongqing. (C1 nature; C2 traditional culture; C3 economy; C4 society; C5 intelligence).

**Figure 8:**
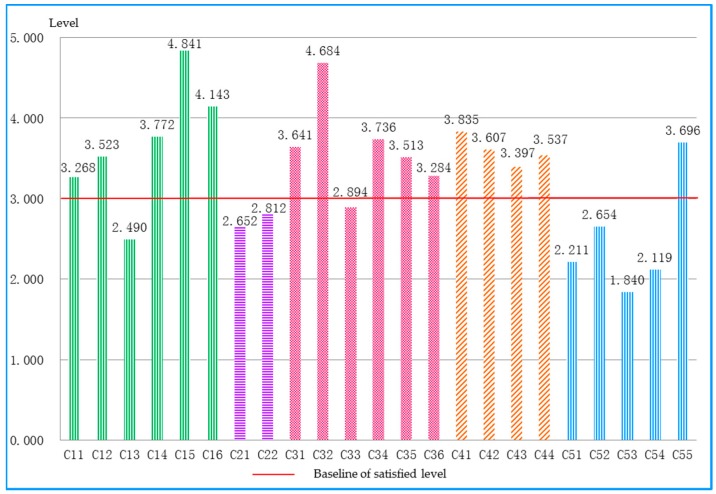
The sustainable performance of the basic indicators in Chongqing.

The sustainable performances for the five dimensions are illustrated in Figure 7. It was discovered that, in the five dimensions, the economic dimension (C3) achieved the top level, while traditional culture (C2) receives the lowest level and intelligence (C5) was the second lowest level. Through the analysis of basic indicators performances as shown in Figure 8, it was discovered that indicator performance of water network denseness index (C13), national material culture heritage (C21), national intangible cultural heritage (C22), urban-rural income ratio (C33), full−time equivalent of Research and Development (R&D) personnel by region (C51), the R&D expenditure input intensity by region (C52), scientific papers issued (C53), and inventions (C54) were below the medium level of 3.0. These results indicate the development of Chongqing had the following characteristics: (1) the ecological environment pressure has increased in Chongqing; (2) Chongqing lacked historical and cultural protection in the process of social development; (3) the economic development of Chongqing has made great achievements, however, the large rural area and population were still the primary barriers to economic construction in Chongqing; (4) the imbalanced urban-rural development was the contradiction facing Chongqing’s society construction; (5) the possible reasons for the low level of intelligence indicators could be the technological innovation ability of the enterprises was weak, and the investment in science and technology was not enough. 

### 5.2. Measures to Improve the Sustainable Urbanization Performance

Traditional urbanization caused many the contradictions of urbanization development. Hence, taking corresponding measures is an important task in the transition from traditional urbanization to sustainable urbanization. The measures are proposed using five themes. 

The first theme is ecological environment protection. Urban planners should alleviate the negative effects of urban development, which cannot be done at the expense of the environment [58]. The increasingly tense water resources and environmental pollution increase the pressure on Chongqing. Therefore, it is necessary to enhance the comprehensive treatment of pollution, and increase the efficiency of resource and energy use.

The second theme is traditional cultural construction. The city is the carrier of culture, and culture has an impact on city’s form and internal quality. Chongqing’s construction should enhance the charm of history and culture, excavate the intangible cultural heritage of the diverse folk and strengthen the culture’s heritage and protection. 

The third theme is optimization of the urban industrial structure. One way is to upgrade traditional industries and eliminate backward production capacity. Another is to adapt to the transformation and upgrade requirements of the manufacturing industry and form a service economy based on the industrial structure. Additionally, it is necessary to prompt the rural economy to eliminate excessive income gaps [59].

The fourth theme is to improve the mechanism of urban rural development. Chongqing is a municipality with the situation of big city and big village, which has the typical rural-urban dual economic structure. This is reflected mainly in the urban and rural household registration barriers, which causes an unbalanced distribution of resources [60,61]. Urban rural development needs to be further strengthened through the elimination of the urban-rural dual structure, such as lifting Hukou restrictions and tightening land regulations [62]. Urban policymakers should balance the allocation of public resources so farmers can participate in the process of urbanization and share the fruits of modernization. 

The fifth theme is intelligence construction. One measure is to strengthen the investment intensity of research and development activities. The other is to coordinate the utilization of information resources and intelligence assets, and push forward the new generation of information technology innovation applications such as cloud computing, big data and more.

## 6. Conclusions

To accurately evaluate sustainable urbanization performance, a comprehensive and reasonable indicator system and an effective method are necessary. This research developed a holistic framework based on an “origin” and “modernization” perspectives for accurately evaluating the sustainable urbanization performance. The multilevel extension assessment method and the AHP method were utilized to complete the evaluation. A case study of Chongqing City in China demonstrated the process of using a holistic framework and evaluation method. The results indicate that Chongqing has a medium level of sustainable urbanization. The city is considered to have a sustainable urbanization performance where “origin” performance is medium and the performance of “modernization” is good, while they are uncoordinated. The case study reveals that the proposed framework and methods are effective theoretical bases for guiding urban managers to make decisions. The sustainable urbanization framework based on “origin” and “modernization” perspectives enriches the relevant research theories of sustainable urbanization development, and provides a reference for the development mode of sustainable urbanization. The multilevel extension method can overcome many of the shortcomings of traditional methods and can be applied to other cities. Limitations of this study are valuable for further research. More representative indicators should be improved in the index system of sustainable urbanization. Additionally, more sample cities should be analyzed.

## Figures and Tables

**Table 1:** Index system of the sustainable urbanization performance evaluation.

Comprehensive indicators-sustainable urbanization performance (U)	**Criteria Layer**	**Dimension Layer**	**Index Layer**
	Origin (A1)	Nature (C1)	C11 Biological richness index
			C12 Vegetation coverage index
			C13 Water network denseness index
			C14 Land stress index
			C15 Pollution load index
			C16 Rocky desertification index
		Traditional culture (C2)	C21 National material culture heritage (unit)
			C22 National intangible cultural heritage (unit)
	Modernization (A2)	Economy (C3)	C31 Real GDP per capita (10,000 Yuan)
			C32 Annual GDP growth rates (%)
			C33 Urban-rural income ratio (%)
			C34 The added value of the tertiary industry shares of GDP (%)
			C35 Per capita disposable income (10,000 Yuan)
			C36 Per capita consumption expenditure of all residents (10,000 Yuan)
		Society (C4)	C41 The number of students on campus per 100,000 persons (Person)
			C42 Average life expectancy (Years old)
			C43 Urbanization rate (%)
			C44 Urban basic public service capacity
		Intelligence (C5)	C51 Full-time equivalent of research and development (R&D) personnel (10,000 man-years)
			C52 The research and development (R&D) expenditure input intensity (%)
			C53 Scientific papers issued (10,000 Piece)
			C54 Inventions (Piece)
			C55 Information development index

**Table 2:** The scale of preference between two parameters in AHP.

Value Meaning	Score
i is equally important to j	1
i is weakly more important to j	3
i is strongly important to j	5
i is very strongly important to j	7
i is absolutely more important to j	9
Intermediate values	2, 4, 6, 8

**Table 3:** The value of random consistency index (RI).

*n*	1	2	3	4	5	6	7	8	9	10
RI	0	0	0.525	0.882	1.115	1.252	1.341	1.404	1.452	1.484

**Table 4:** The judgment matrix and weight based on U.

U	A1	A2	$\lambda_{max}$	Weight	$CI=\left(\lambda_{max}-n\right)/\left(n-1\right)$
A1	1	1	2.005	0.500	CI = 0.005 < 0.1 Uniform convergence
A2	1	1		0.500	

**Table 5:** The judgment matrix and weight based on A1.

A1	C1	C2	$\lambda_{max}$	Weight	$CI=\left(\lambda_{max}-n\right)/\left(n-1\right)$
C1	1	1	2.005	0.500	CI = 0.005 < 0.1 Uniform convergence
C2	1	1		0.500	

**Table 6:** The judgment matrix and weight based on A2.

A2	C3	C4	C5	$\lambda_{max}$	Weight	$CI=\left(\lambda_{max}-n\right)/\left(n-1\right)$
C3	1	1	1/2	3.104	0.413	CI = 0.052 < 0.1 Uniform convergence
C4	1	1	1		0.327	
C5	2	1	1		0.260	

**Table 7:** The judgment matrix and weight based on C1.

C1	C11	C12	C13	C14	C15	C16	$\lambda_{max}$	Weight	$CI=\left(\lambda_{max}-n\right)/\left(n-1\right)$
C11	1	1	1/2	1/3	1/3	1/2	6.320	0.267	CI = 0.064 < 0.1 Uniform convergence
C12	1	1	1	1/2	1/4	1/3		0.264	
C13	2	1	1	1/2	1	1/2		0.161	
C14	3	2	2	1	3	1		0.080	
C15	3	4	1	1/3	1	1/3		0.148	
C16	2	3	2	1	3	1		0.080	

**Table 8:** The judgment matrix and weight based on C2.

C2	C21	C22	$\lambda_{max}$	Weight	$CI=\left(\lambda_{max}-n\right)/\left(n-1\right)$
C21	1	1	2.005	0.500	CI = 0.005 < 0.1 Uniform convergence
C22	1	1		0.500	

**Table 9:** The judgment matrix and weight based on C3.

C3	C31	C32	C33	C34	C35	C36	$\lambda_{max}$	Weight	$CI=\left(\lambda_{max}-n\right)/\left(n-1\right)$
C31	1	1/2	1/3	1/2	1/4	1/6	6.240	0.345	CI = 0.048 < 0.1 Uniform convergence
C32	2	1	1/3	1	1/4	1/3		0.232	
C33	1/3	3	1	1/2	1/3	1/4		0.166	
C34	2	1	2	1	1/2	1/2		0.137	
C35	4	4	3	2	1	1		0.062	
C36	6	3	4	2	1	1		0.058	

**Table 10:** The judgment matrix and weight based on C4.

C4	C41	C42	C43	C44	$\lambda_{max}$	Weight	$CI=\left(\lambda_{max}-n\right)/\left(n-1\right)$
C41	1	1	1/2	3	4.024	0.190	CI = 0.008 < 0.1 Uniform convergence
C42	1	1	1/2	3		0.190	
C43	2	2	1	4		0.105	
C44	1/3	1/3	1/4	1		0.515	

**Table 11:** The judgment matrix and weight based on C5.

C5	C51	C52	C53	C54	C55	$\lambda_{max}$	Weight	$CI=\left(\lambda_{max}-n\right)/\left(n-1\right)$
C51	1	1	2	2	3	5.268	0.104	CI = 0.067 < 0.1 Uniform convergence
C52	1	1	1/2	1/2	4		0.163	
C53	1/2	2	1	1	5		0.118	
C54	1/2	2	1	1	5		0.118	
C55	1/3	1/4	1/5	1/5	1		0.497	

**Table 12:** The classical field, segment field, actual value and data source.

Indicator	$N_{j}\;\mathrm{Classical}\;\mathrm{Field}$					$N_{P}\;\mathrm{Segment}\;\mathrm{Field}$	Actual Value	Data Source
	$N_{1}\;\mathrm{Excellent}$	$N_{2}\;\mathrm{Good}$	$N_{3}\;\mathrm{Medium}$	$N_{4}\;\mathrm{Fair}$	$N_{5}\;\mathrm{Poor}$			
C11	<75, 100>	<55, 75>	<35, 55>	<20, 35>	<0, 20>	<0, 100>	51.10	[49]
C12	<75, 100>	<55, 75>	<35, 55>	<20, 35>	<0, 20>	<0, 100>	55.39	[49]
C13	<50, 100>	<30, 50>	<10, 30>	<5, 10>	<0, 5>	<0, 100>	16.47	[49]
C14	<0, 10>	<10, 30>	<30, 40>	<40, 60>	<60, 100>	<0, 100>	21.63	[49]
C15	<61.3, 100>	<27.6, 61.3>	<9.7, 27.6>	<2.6, 9.7>	<0, 2.6>	<0, 100>	91.85	[49]
C16	<0, 20>	<20, 30>	<30, 40>	<40, 60>	<60,100>	<0, 100>	17.35	[49]
C21	<200, 500>	<60, 200>	<40, 60>	<20, 40>	<0, 20>	<0, 500>	55.00	[53]
C22	<100, 170>	<45, 100>	<30, 45>	<10, 30>	<0, 10>	<0, 170>	41.00	[54]
C31	<7, 12>	<5, 7>	<3, 5>	<2, 3>	<0, 2>	<0, 12>	5.23	[47]
C32	<9, 12>	<7, 9>	<5, 7>	<3, 5>	<0, 3>	<0, 12>	11.00	[52]
C33	<0, 1>	<1, 2>	<2, 3>	<3, 4>	<4, 5>	<0, 5>	2.59	[47]
C34	<50, 100>	<40, 50>	<30, 40>	<20, 30>	<0, 20>	<0, 100>	47.70	[47]
C35	<3, 5>	<2, 3>	<1, 2>	<0.5, 1>	<0, 0.5>	<0, 5>	2.01	[47]
C36	<2.5, 3.5>	<1.5, 2.5>	<1, 1.5>	<0.5, 1>	<0, 0.5>	<0, 3.5>	1.51	[47]
C41	<3500, 5500>	<2500, 3500>	<2000, 2500>	<1000, 2000>	<0, 1000>	<0, 5500>	3071	[47]
C42	<80, 100>	<75, 80>	<70, 75>	<60, 70>	<0, 60>	<0, 100>	75.70	[52]
C43	<80, 100>	<60, 80>	<50, 60>	<30, 50>	<0, 30>	<0, 100>	60.94	[47]
C44	<65, 100>	<55, 65>	<50, 55>	<45, 50>	<0, 45>	<0, 100>	61.80	[50]
C51	<50, 60>	<20, 50>	<5, 20>	<1, 5>	<0, 1>	<0, 60>	6.15	[48]
C52	<5, 6.5>	<2, 5>	<1, 2>	<0.5, j1>	<0, 0.5>	<0, 6.5>	1.57	[48]
C53	<1, 6>	<0.6, 1>	<0.3, 0.6>	<0.1, 0.3>	<0, 0.1>	<0, 6>	0.18	[48]
C54	<1, 1.5>	<0.2, 1>	<0.06, 0.2>	<0.01, 0.06>	<0, 0.01>	<0, 1.5>	0.05	[48]
C55	<80, 100>	<70, 80>	<60, 70>	<50, 60>	<0, 50>	<0, 100>	72.18	[51]

**Table 13:** The correlations and evaluation results.

Indicator	Excellent	Good	Medium	Fair	Poor	Max	$j_{0}$	Grade	$j^{*}$
U	−0.345	−0.066	−0.016	−0.296	−0.453	−0.016	3	Medium	3.411
A1	−0. 459	−0.130	0.077	−0.313	−0.470	0.077	3	Medium	3.195
A2	−0.230	−0.001	−0.109	−0.279	−0.436	−0.001	4	Good	3.613
C1	−0.262	−0.181	−0.086	−0.411	−0.530	−0.086	3	Medium	3.648
C2	−0.656	−0.080	0.239	−0.216	−0.411	0.239	3	Medium	2.722
C3	−0.126	−0.115	−0.152	−0.399	−0.499	−0.115	4	Good	3.855
C4	−0.131	0.278	−0.122	−0.239	−0.366	0.278	4	Good	3.789
C5	−0.521	−0.170	−0.025	−0.140	−0.423	−0.025	3	Medium	2.830
C11	−0.328	−0.074	0.195	−0.248	−0.389	0.195	3	Medium	3.268
C12	−0.305	0.020	−0.009	−0.314	−0.442	0.020	4	Good	3.523
C13	−0.671	−0.451	0.323	−0.282	−0.410	0.323	3	Medium	2.490
C14	−0.350	0.418	−0.279	−0.459	−0.639	0.418	4	Good	3.772
C15	0.211	−0.789	−0.887	−0.910	−0.916	0.211	5	Excellent	4.841
C16	0.132	−0.132	−0.422	−0.566	−0.711	0.132	5	Excellent	4.143
C21	−0.721	−0.070	0.211	−0.220	−0.391	0.211	3	Medium	2.652
C22	−0.590	−0.089	0.267	−0.212	−0.431	0.267	3	Medium	2.812
C31	−0.253	0.115	−0.042	−0.299	−0.382	0.115	4	Good	3.641
C32	0.333	−0.667	−0.800	−0.857	−0.889	0.333	5	Excellent	4.684
C33	−0.398	−0.197	0.410	−0.145	−0.369	0.410	3	Medium	2.894
C34	−0.046	0.230	−0.139	−0.271	−0.367	0.230	4	Good	3.736
C35	−0.330	0.010	−0.005	−0.334	−0.429	0.010	4	Good	3.513
C36	−0.396	0.010	−0.007	−0.252	−0.401	0.010	4	Good	3.284
C41	−0.150	0.429	−0.190	−0.306	−0.460	0.429	4	Good	3.835
C42	−0.150	0.140	−0.028	−0.190	−0.393	0.140	4	Good	3.607
C43	−0.328	0.047	−0.023	−0.219	−0.442	0.047	4	Good	3.397
C44	−0.077	0.320	−0.151	−0.236	−0.305	0.320	4	Good	3.537
C51	−0.877	−0.693	0.077	−0.158	−0.456	0.077	3	Medium	2.211
C52	−0.686	−0.215	0.430	−0.266	−0.405	0.430	3	Medium	2.654
C53	−0.820	−0.700	−0.400	0.400	−0.308	0.400	2	Fair	1.840
C54	−0.950	−0.750	−0.167	0.200	−0.444	0.200	2	Fair	2.119
C55	−0.219	0.218	−0.073	−0.305	−0.444	0.218	4	Good	3.696
